# Supplementary material for: Multivariate classification of multichannel long-term electrophysiology data identifies different sleep stages in fruit flies
Source: bioRxiv. 2023 Jun 13:2023.06.12.544704. Preprint. [Version 1] doi: 10.1101/2023.06.12.544704 (PMC10312633; doi:10.1101/2023.06.12.544704)
Supplement: Supplement 1 [file NIHPP2023.06.12.544704v1-supplement-1.pdf]

bioRxiv preprint doi: https://doi.org/10.1101/2023.06.12.544704; this version posted June 13, 2023. The copyright holder for this preprint (which was not certified by peer review) is the author/funder, who has granted bioRxiv a license to display the preprint in perpetuity. It is made available under aCC-BY 4.0 International license.

Supplementary material

Suppl Table 1: Model comparison - Left antenna

**Model**	**Parameters**	**Log-likelihood**	**Pr(>χ2)**
Null	Fixed: mean, Random: fly ID	-106.53	-
epoch	Fixed: time_label, Random: fly ID	**-73.58**	<0.001

Suppl Table 2: Type III analysis of variance with Satterthwaite's method of the winning model (Epoch) - Left antenna

**Model elements**	**Sum Sq**	**Mean Sq**	**NumDF**	**DenDF**	**F value**	**Pr(>F)**
Epoch	4.5527	1.1382	4	1090	16.985	<0.001

Suppl Table 3: Model comparison - Right antenna

**Model**	**Parameters**	**Log-likelihood**	**Pr(>χ2)**
Null	Fixed: mean, Random: fly ID	-68.415	-
epoch	Fixed: time_label, Random: fly ID	**-44.468**	<0.001

Suppl Table 4: Type III analysis of variance with Satterthwaite's method of the winning model (Epoch) - Right antenna

**Model elements**	**Sum Sq**	**Mean Sq**	**NumDF**	**DenDF**	**F value**	**Pr(>F)**
Epoch	3.1004	0.7751	4	1125	12.232	<0.001

bioRxiv preprint doi: https://doi.org/10.1101/2023.06.12.544704; this version posted June 13, 2023. The copyright holder for this preprint (which was not certified by peer review) is the author/funder, who has granted bioRxiv a license to display the preprint in perpetuity. It is made available under aCC-BY 4.0 International license.

Suppl Table 5: Model comparison - PEs

Model	Parameters	Log-likelihood	Pr(>χ2)
Null	Fixed: mean, Random: fly ID	-301.09	-
epoch	Fixed: time_label, Random: fly ID	**-207.25**	<0.001

Suppl Table 6: Type III analysis of variance with Satterthwaite's method of the winning model (Epoch) - PEs

Model elements	Sum Sq	Mean Sq	NumDF	DenDF	F value	Pr(>F)
Epoch	20.877	5.2192	4	795	52.923	<0.001

Suppl Table 7: Model comparison - LFP power spectrum

Model	Parameters	Log-likelihood	Pr(>χ2)
Null	Fixed: mean, Random: fly ID	-68117	-
Epoch	Fixed: epoch, Random: fly ID	-67941	<0.001
Channel	Fixed: channel, Random: fly ID	-52391	<0.001
Epoch-Channel	Fixed: epoch*channel, Random: fly ID	**-51593**	<0.001

bioRxiv preprint doi: https://doi.org/10.1101/2023.06.12.544704; this version posted June 13, 2023. The copyright holder for this preprint (which was not certified by peer review) is the author/funder, who has granted bioRxiv a license to display the preprint in perpetuity. It is made available under aCC-BY 4.0 International license.

Suppl Table 8: Type III analysis of variance with Satterthwaite's method of the winning model (Epoch-Channel) - LFP power spectrum

Model elements	Sum Sq	Mean Sq	NumDF	DenDF	F value	Pr(>F)
Epoch	8476	2119	4	22582	378.025	<0.001
Channel	112004	56002	2	22580	9990.441	<0.001
Epoch:Channel	796	100	8	22580	17.756	<0.001

Suppl Table 9: Model comparison - PEs LFP dataset

Model	Parameters	Log-likelihood	Pr(>χ2)
Null	Fixed: mean, Random: fly ID	-4.5588	-
time_label	Fixed: time_label, Random: fly ID	**15.0632**	<0.001

Suppl Table 10: Type III analysis of variance with Satterthwaite's method of the winning model (time_label) - PEs LFP dataset

Model elements	Sum Sq	Mean Sq	NumDF	DenDF	F value	Pr(>F)
time_label	1.2145	0.20241	6	41	9.6039	<0.001

bioRxiv preprint doi: https://doi.org/10.1101/2023.06.12.544704; this version posted June 13, 2023. The copyright holder for this preprint (which was not certified by peer review) is the author/funder, who has granted bioRxiv a license to display the preprint in perpetuity. It is made available under aCC-BY 4.0 International license.

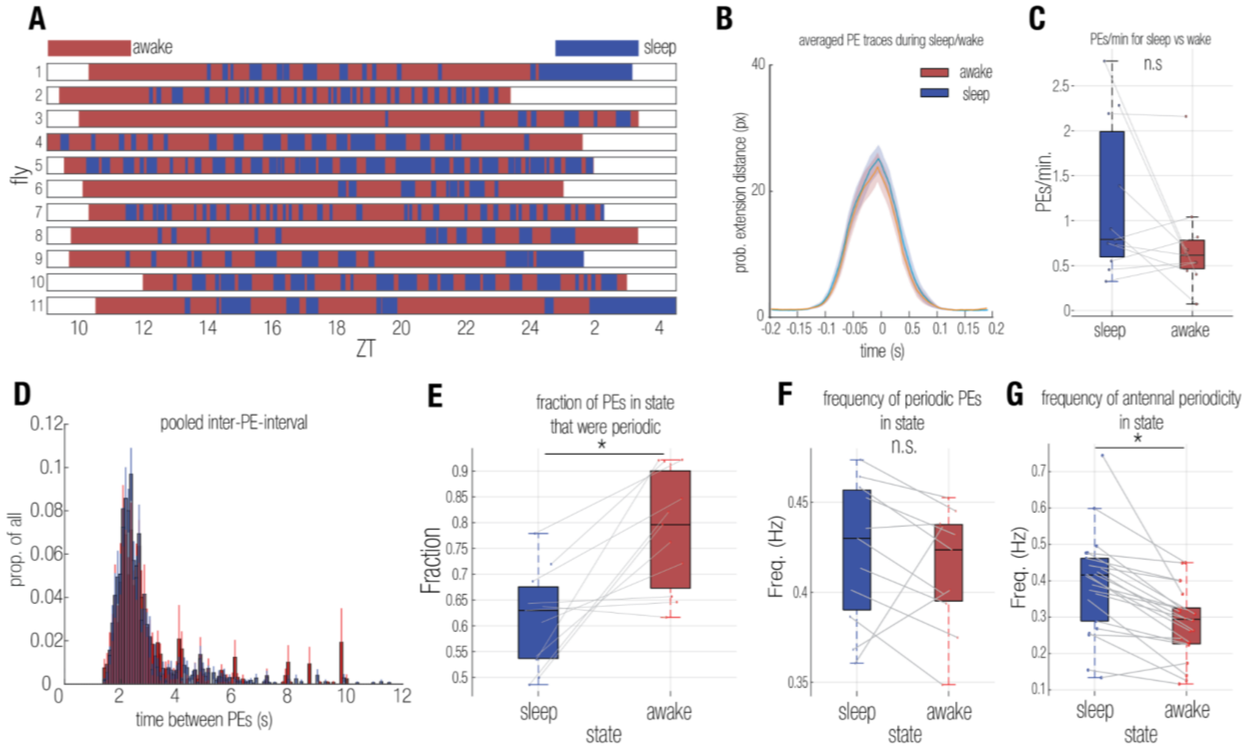

Supplementary Figure 1: Additional metrics of proboscis activity during sleep and wake. A) Representation of the distribution of sleep (Blue) and wake (Red) across N=11 recorded individuals over the course of time. B) Averaged timecourse of proboscis extension distance from resting during a single event for sleep (Blue) and wake (Red). C) Comparison of proboscis extension rates during sleep and wake (n.s. ; Student's T-test). D) Histogram of the distribution of times between PEs during sleep (Blue) and wake (Red). E) Comparison of the fraction of PEs that were periodic versus isolated for sleep and wake ($p < 0.05$; Student's T-test). F) Comparison of the average frequency of PE periodicity across sleep and wake (n.s.; Student's T-test). G) As with F, for antennal periodicity ($p < 0.05$; Student's T-test).

bioRxiv preprint doi: https://doi.org/10.1101/2023.06.12.544704; this version posted June 13, 2023. The copyright holder for this preprint (which was not certified by peer review) is the author/funder, who has granted bioRxiv a license to display the preprint in perpetuity. It is made available under aCC-BY 4.0 International license.

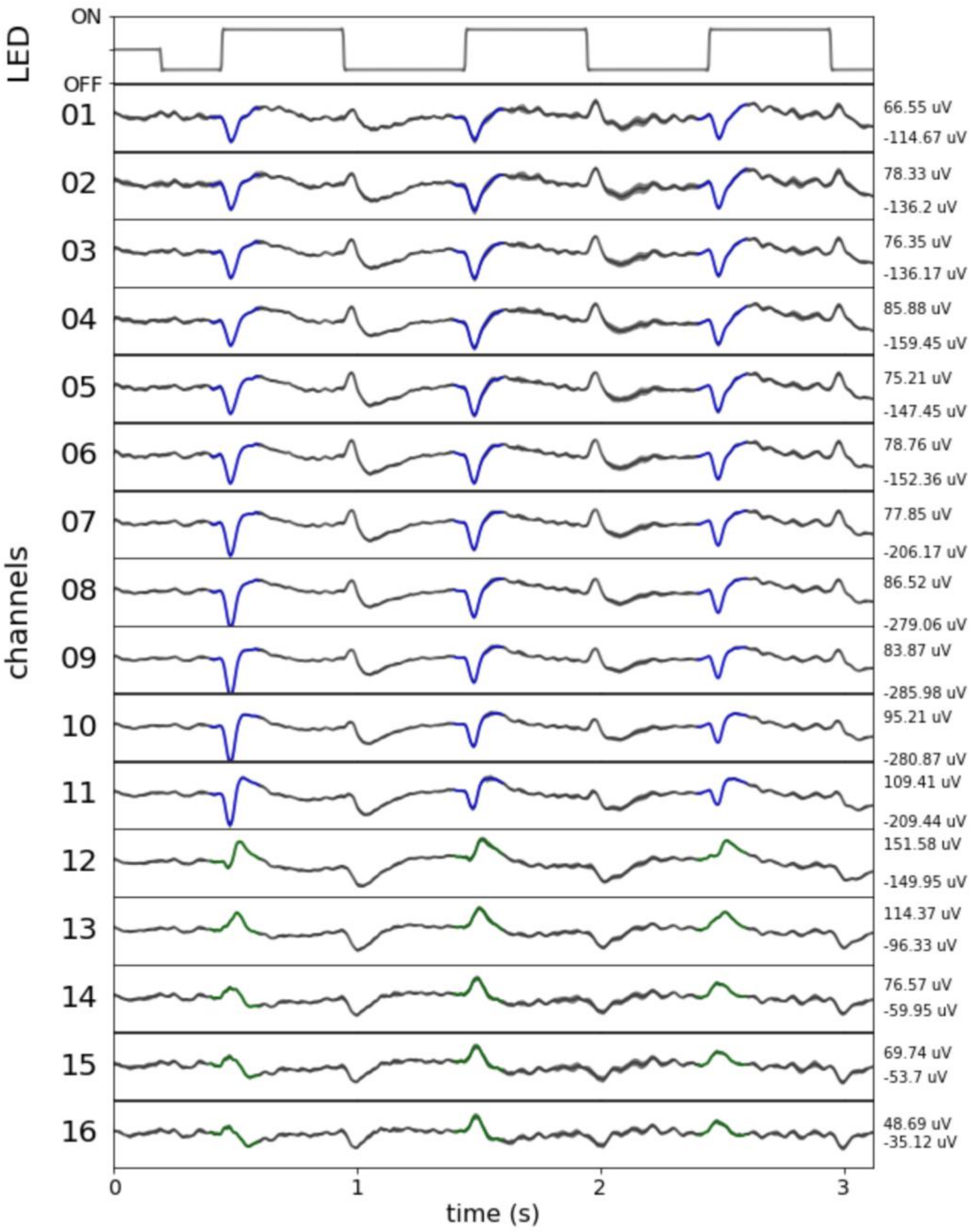

Supplementary Figure 2: Electrode insertion depth was controlled by using a polarity reversal method. In this example fly, the change in LED stimulation (OFF to ON) stage, coincides with a LFP deflection. The LFP deflection changes from positive (12th channel) to negative (11th channel). The LFP amplitude depicted here is based on an average of 5 trials, with the shaded region representing the standard error.

bioRxiv preprint doi: https://doi.org/10.1101/2023.06.12.544704; this version posted June 13, 2023. The copyright holder for this preprint (which was not certified by peer review) is the author/funder, who has granted bioRxiv a license to display the preprint in perpetuity. It is made available under aCC-BY 4.0 International license.

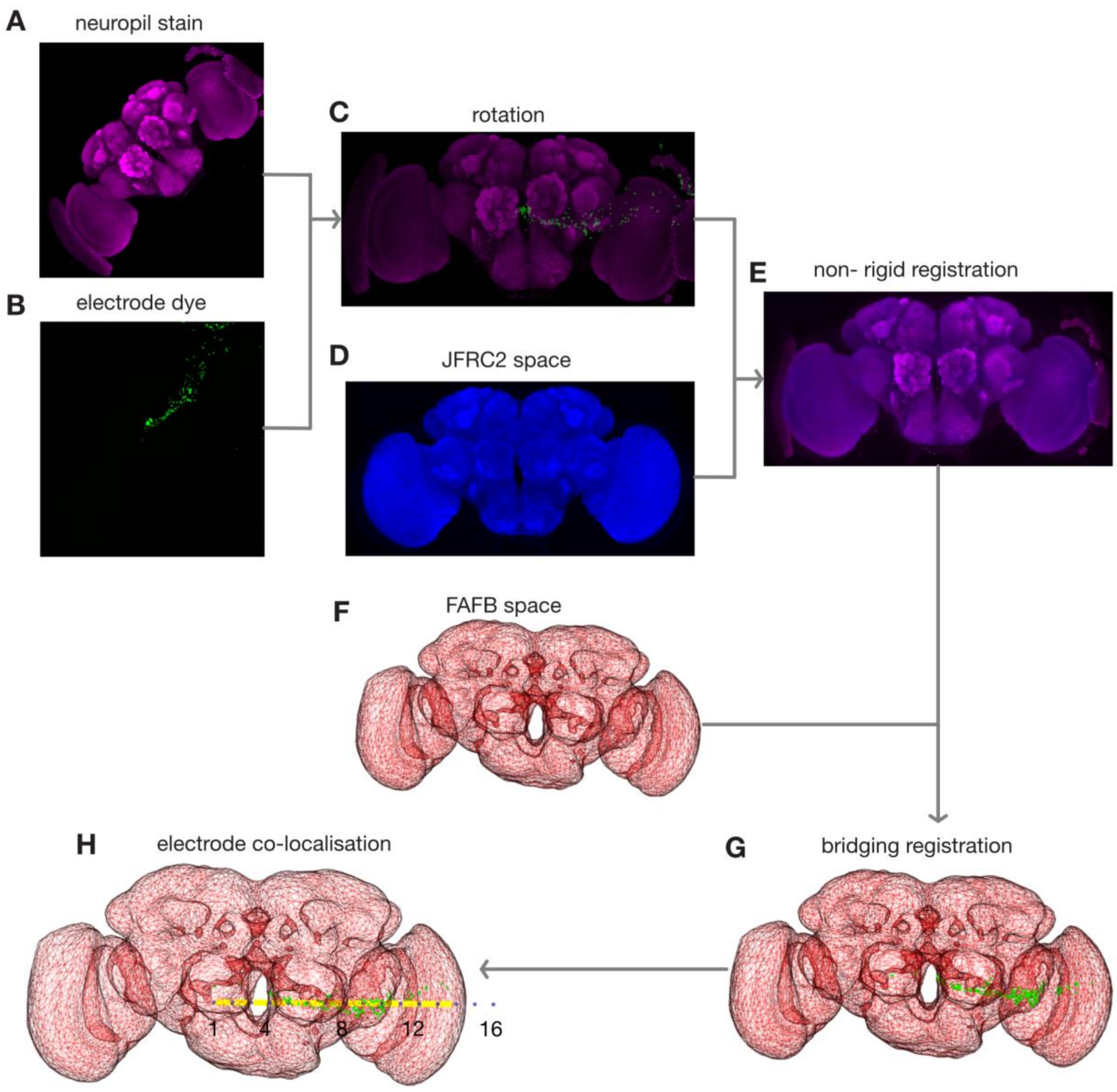

Supplementary Figure 3: Electrode locations were determined using a dye based localisation method. Neuropil stain (A) and electrode dye locations (B) were registered to JFRC2 space (D) via non-rigid registration. Further Bridging registration was used to register to FAFB space (F), the registration templates were applied on electrode dye locations (B) to produce co-localisation (H).

bioRxiv preprint doi: https://doi.org/10.1101/2023.06.12.544704; this version posted June 13, 2023. The copyright holder for this preprint (which was not certified by peer review) is the author/funder, who has granted bioRxiv a license to display the preprint in perpetuity. It is made available under aCC-BY 4.0 International license.

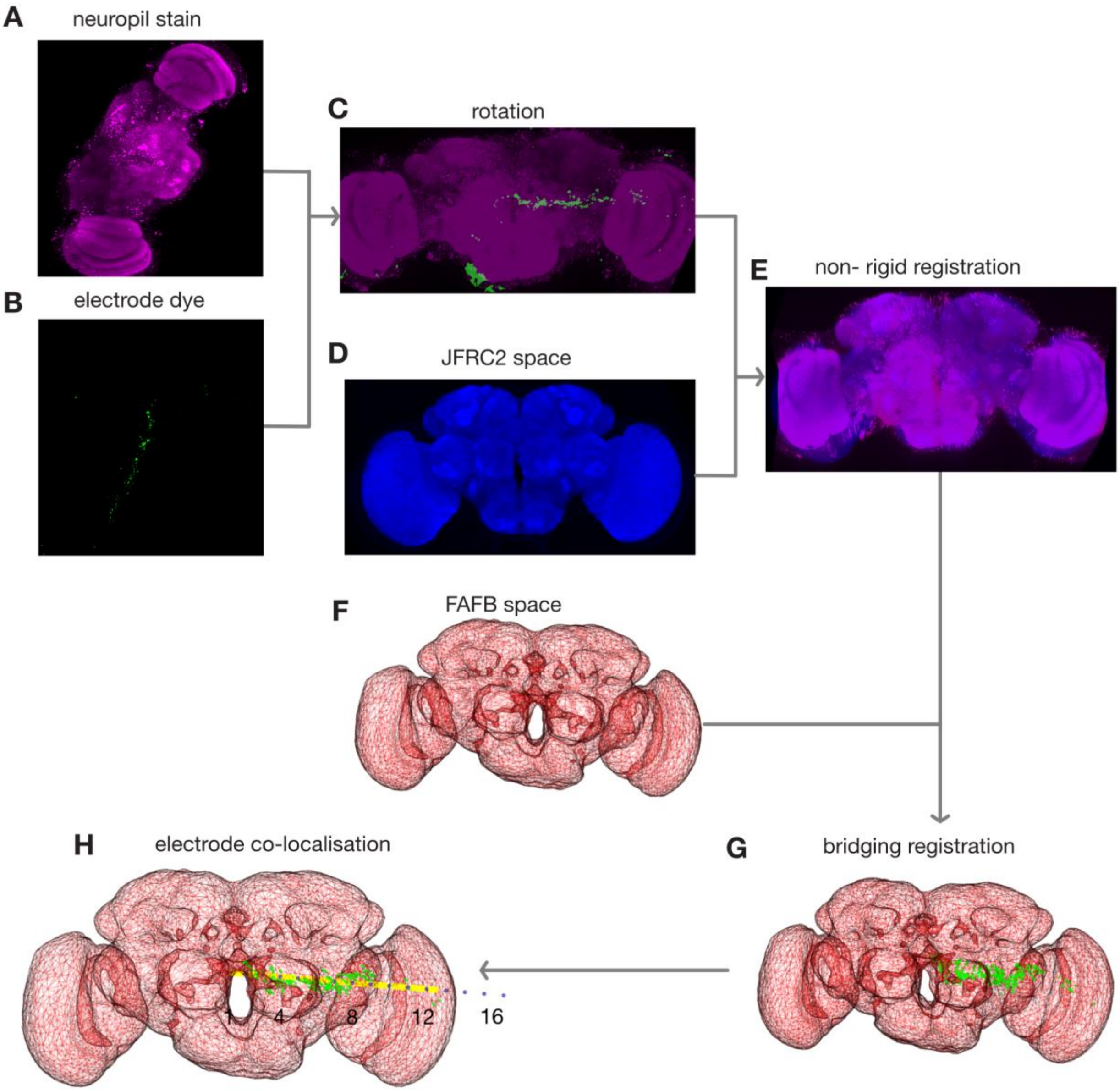

Supplementary Figure 4: Electrode locations were determined using a dye based localisation method. Neuropil stain (A) and electrode dye locations (B) were registered to JFRC2 space (D) via non-rigid registration. Further Bridging registration was used to register to FAFB space (F), the registration templates were applied on electrode dye locations (B) to produce co-localisation (H).

bioRxiv preprint doi: https://doi.org/10.1101/2023.06.12.544704; this version posted June 13, 2023. The copyright holder for this preprint (which was not certified by peer review) is the author/funder, who has granted bioRxiv a license to display the preprint in perpetuity. It is made available under aCC-BY 4.0 International license.

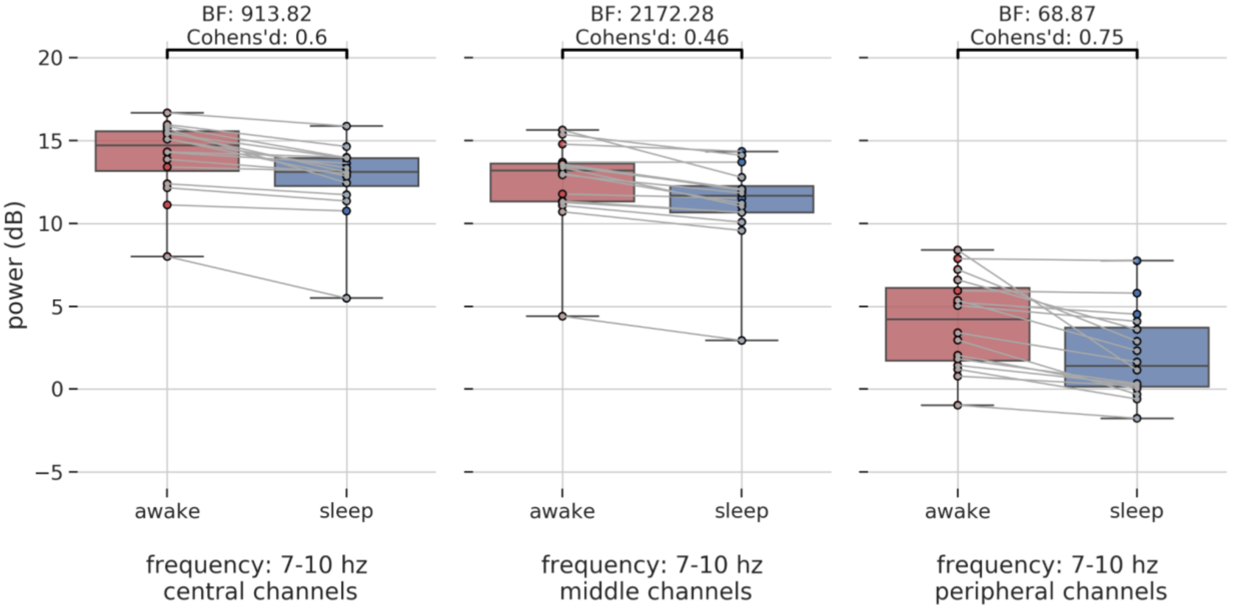

Supplementary Figure 5: Power differences across central, middle, peripheral channels in the frequency bands of 7-10 Hz.

bioRxiv preprint doi: https://doi.org/10.1101/2023.06.12.544704; this version posted June 13, 2023. The copyright holder for this preprint (which was not certified by peer review) is the author/funder, who has granted bioRxiv a license to display the preprint in perpetuity. It is made available under aCC-BY 4.0 International license.

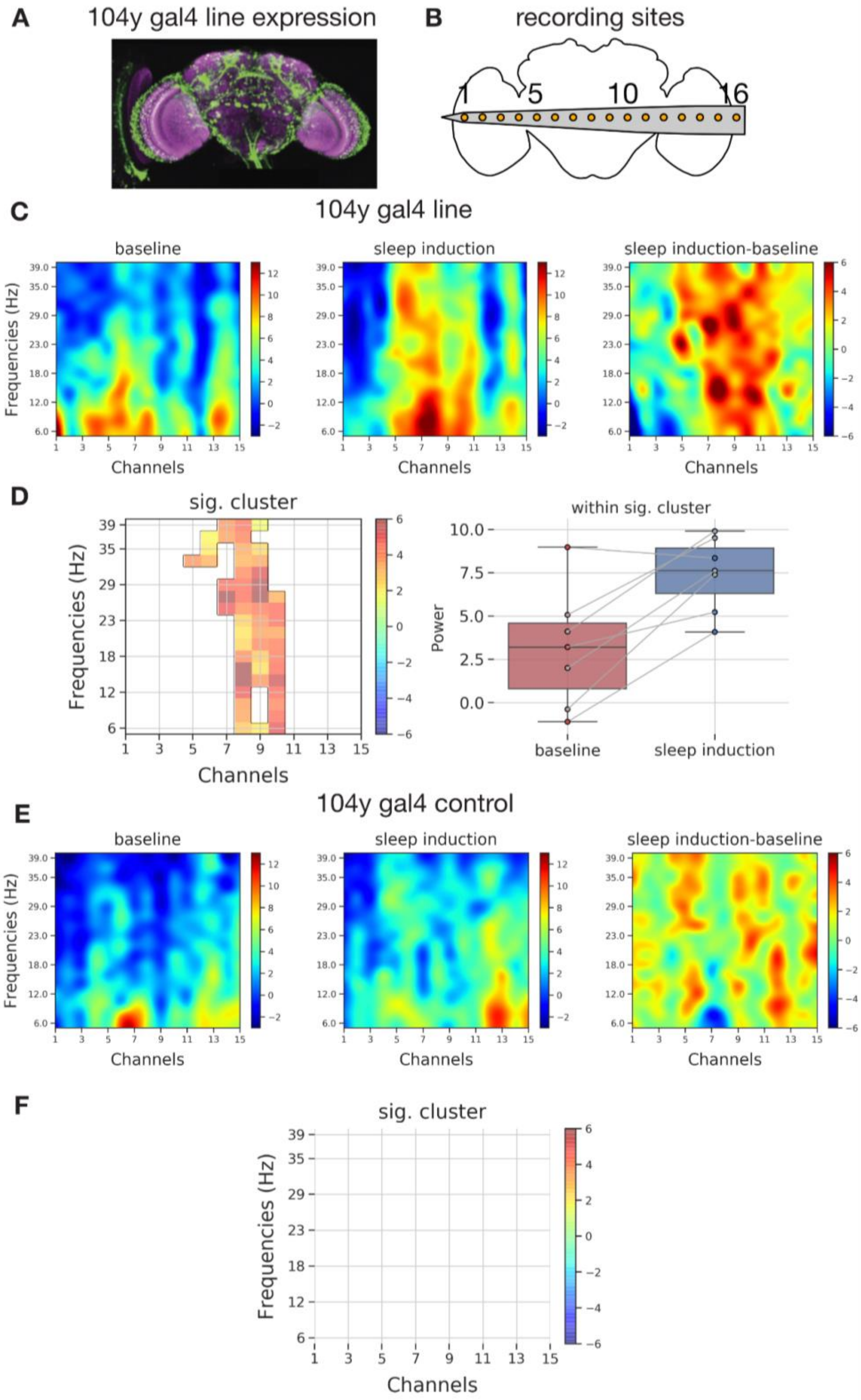

1443 **Supplementary Figure 6:** Spectral differences in thermogenetically induced sleep recorded using full brain probe.

1444

bioRxiv preprint doi: https://doi.org/10.1101/2023.06.12.544704; this version posted June 13, 2023. The copyright holder for this preprint (which was not certified by peer review) is the author/funder, who has granted bioRxiv a license to display the preprint in perpetuity. It is made available under aCC-BY 4.0 International license.

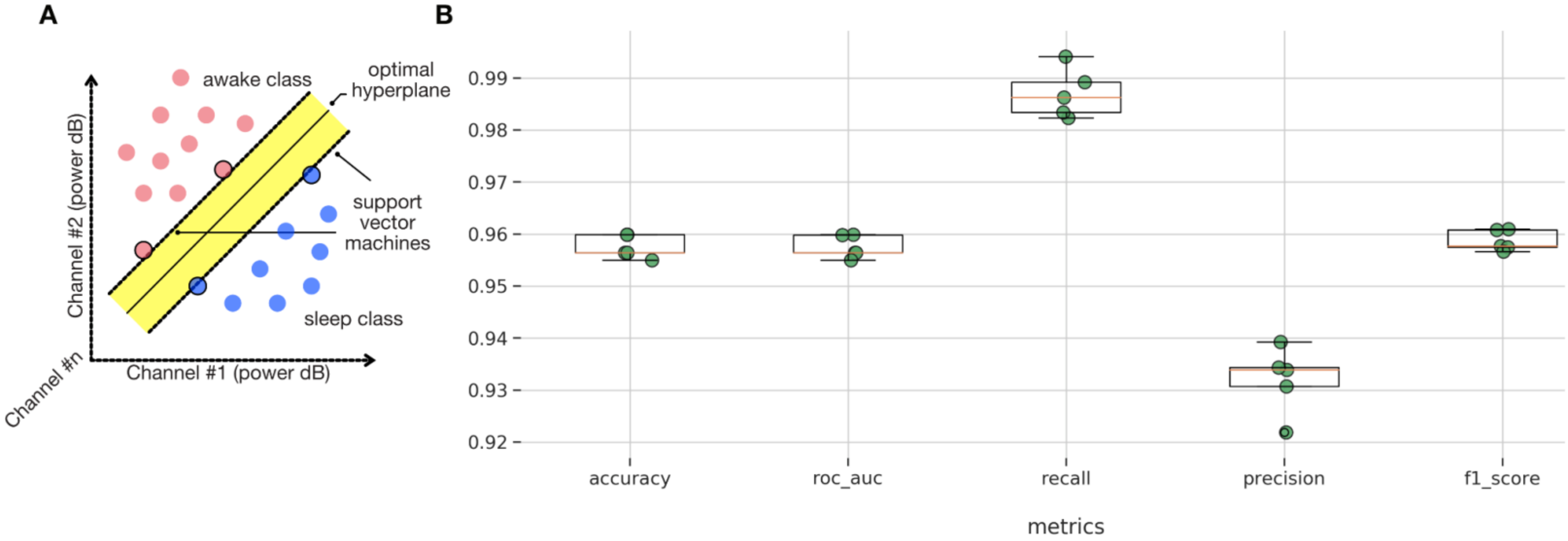

Supplementary Figure 7: A) Schematic indicating the optimal separation of awake and sleep classes using classifiers based on support vector machines. B) SVM based classifier performance across different metrics based on 5 different train/test data splits.

bioRxiv preprint doi: https://doi.org/10.1101/2023.06.12.544704; this version posted June 13, 2023. The copyright holder for this preprint (which was not certified by peer review) is the author/funder, who has granted bioRxiv a license to display the preprint in perpetuity. It is made available under aCC-BY 4.0 International license.

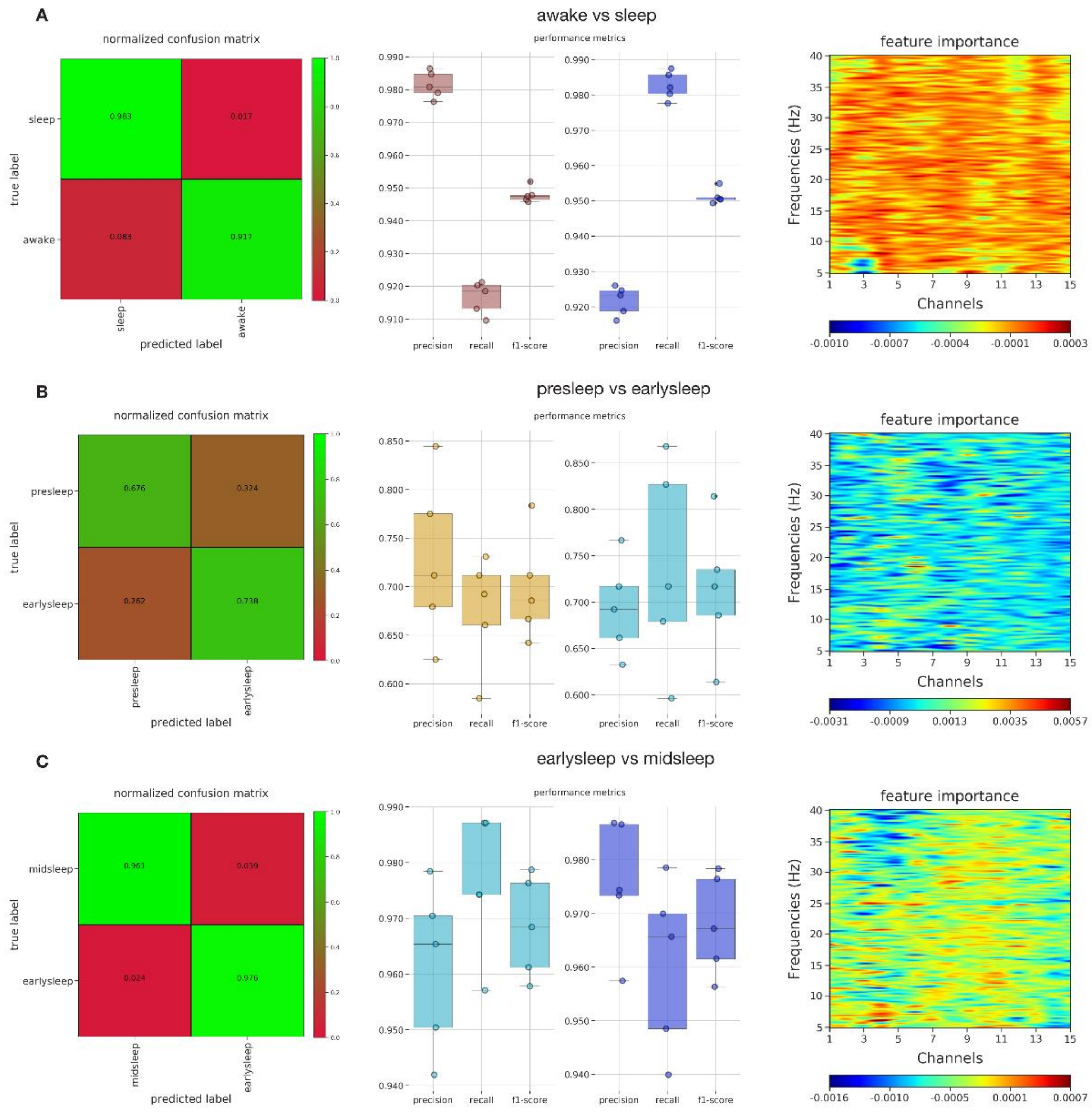

bioRxiv preprint doi: https://doi.org/10.1101/2023.06.12.544704; this version posted June 13, 2023. The copyright holder for this preprint (which was not certified by peer review) is the author/funder, who has granted bioRxiv a license to display the preprint in perpetuity. It is made available under aCC-BY 4.0 International license.

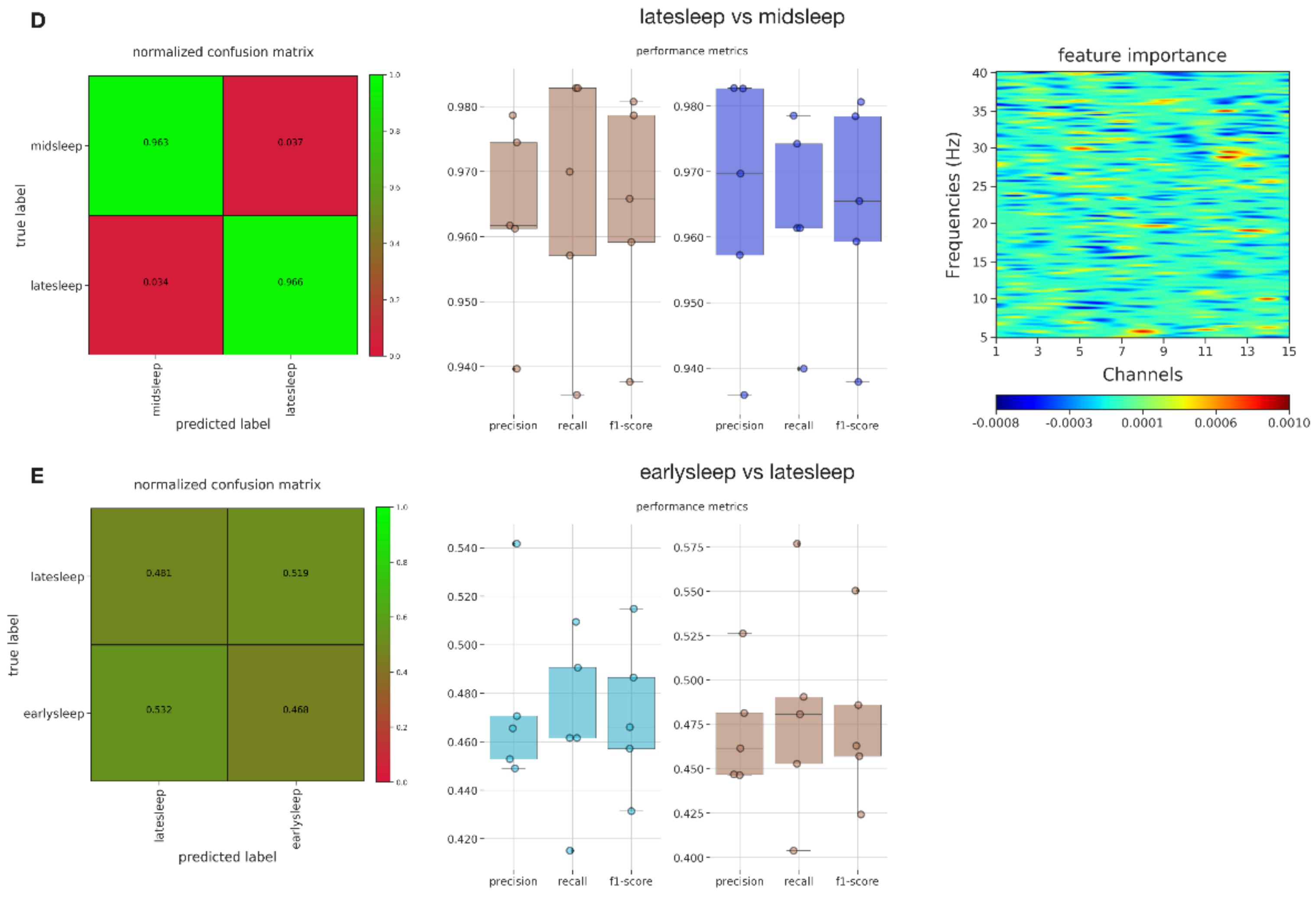

Supplementary Figure 8: A) Feature importance of the multiclass classifier (reduced to awake vs sleep) indicates an ROI across all channels and almost all frequency bands as critically important. This cross validates the differences in the power spectrum across awake and sleep as shown in Figure 4D. B,C,D,E) Feature importance of multiclass classifier for the other categories.
